# Predicting the Development of Diabetes Using the Product of Triglycerides and Glucose: The Chungju Metabolic Disease Cohort (CMC) Study

**DOI:** 10.1371/journal.pone.0090430

**Published:** 2014-02-28

**Authors:** Seung-Hwan Lee, Hyuk-Sang Kwon, Yong-Moon Park, Hee-Sung Ha, Seung Hee Jeong, Hae Kyung Yang, Jin-Hee Lee, Hyeon-Woo Yim, Moo-Il Kang, Won-Chul Lee, Ho-Young Son, Kun-Ho Yoon

**Affiliations:** 1 Department of Internal Medicine, College of Medicine, The Catholic University of Korea, Seoul, Korea; 2 Division of Endocrinology and Metabolism, Department of Internal Medicine, Seoul St. Mary’s Hospital, Seoul, Korea; 3 Division of Endocrinology and Metabolism, Department of Internal Medicine, Yeouido St. Mary’s Hospital, Seoul, Korea; 4 Department of Preventive Medicine, College of Medicine, The Catholic University of Korea, Seoul, Korea; 5 Department of Epidemiology and Biostatistics, Arnold School of Public Health, University of South Carolina, Columbia, South Carolina, United States of America; 6 Clinical Research Coordinating Center, College of Medicine, The Catholic University of Korea, Seoul, Korea; 7 Catholic Institute of U-Healthcare, The Catholic University of Korea, Seoul, Korea; CAEBi, Spain

## Abstract

**Background:**

To determine whether the TyG index, a product of the levels of triglycerides and fasting plasma glucose (FPG) might be a valuable marker for predicting future diabetes.

**Methods:**

A total of 5,354 nondiabetic subjects who had completed their follow-up visit for evaluating diabetes status were selected from a large cohort of middle-aged Koreans in the Chungju Metabolic Disease Cohort study. The risk of diabetes was assessed according to the baseline TyG index, calculated as ln[fasting triglycerides (mg/dL) × FPG (mg/dL)/2]. The median follow-up period was 4.6 years.

**Results:**

During the follow-up period, 420 subjects (7.8%) developed diabetes. The baseline values of the TyG index were significantly higher in these subjects compared with nondiabetic subjects (8.9±0.6 vs. 8.6±0.6; *P*<0.0001) and the incidence of diabetes increased in proportion to TyG index quartiles. After adjusting for age, gender, body mass index, waist circumference, systolic blood pressure, high-density lipoprotein (HDL)-cholesterol level, a family history of diabetes, smoking, alcohol drinking, education level and serum insulin level, the risk of diabetes onset was more than fourfold higher in the highest vs. the lowest quartile of the TyG index (relative risk, 4.095; 95% CI, 2.701–6.207). The predictive power of the TyG index was better than the triglyceride/HDL-cholesterol ratio or the homeostasis model assessment of insulin resistance.

**Conclusions:**

The TyG index, a simple measure reflecting insulin resistance, might be useful in identifying individuals at high risk of developing diabetes.

## Introduction

The incidence and prevalence of type 2 diabetes is increasing rapidly worldwide, and is expected to affect 439 million adults by 2030 [1]–[3]. To overcome the burden of this epidemic, public health strategy should focus on the identification of subjects at high risk of developing diabetes with the aim of prevention and proper intervention. This might lead to favorable outcomes while avoiding unnecessary effort [4]. For this purpose, an easily measurable and widely applicable index with high predictability needs to be developed.

Various risk factors and predictive models have been suggested. In addition to traditional risk factors, inflammatory biomarkers and genetic risk factors are under investigation to improve the prediction of diabetes. However, the additive role of these novel markers is reported to be limited [5]–[8]. Among traditional risk factors, the levels of fasting plasma glucose (FPG) and triglycerides (TG) are well validated for their role in predicting the development of diabetes. Higher FPG levels have been shown to be an independent risk factor for developing type 2 diabetes even when subjects are within the normoglycemic range [9], [10]. Elevations and increases in TG levels over time also enhanced the risk of developing diabetes in various populations [11]–[14].

The TyG index, the product of FPG and TG, is a novel index that has been suggested as a surrogate of insulin resistance in healthy subjects [15], [16]. It was well correlated with the homeostasis model assessment of insulin resistance (HOMA-IR) and total glucose metabolism rates during hyperinsulinemic–euglycemic clamp studies. Given that insulin resistance is the core pathophysiologic mechanism of type 2 diabetes and is already present 1–2 decades before the diagnosis [17], [18], surrogate indices of insulin resistance might aid in the prediction of incident diabetes.

In this study, we aimed to determine whether the TyG index could be a valuable marker for predicting diabetes and to compare it with other insulin resistance indices in a large-scale cohort of middle-aged nondiabetic Koreans.

## Subjects and Methods

### Subjects

The Chungju Metabolic disease Cohort (CMC) study is an ongoing community-based study of metabolic diseases including diabetes, metabolic syndrome and osteoporosis in a population aged 40 years and over living in the rural area of Chungju City, Korea. The first phase (baseline) of the study was performed in 2003–2006. Three hundred thirty-four districts were selected by stratified random cluster sampling and 11,718 subjects participated. These participants were follwed up at 4-year intervals in the second phase of the study (2007–2010) with a follow-up rate of 58%, and the third phase of the study is ongoing (2011–2014). Subjects with known or newly diagnosed diabetes or prediabetes at the baseline study, and those lacking laboratory or anthropometric data were excluded. In this analysis, persons who had completed their follow-up visit at the second phase (n = 3,814) or both second and third phases (n = 1,540) of the study were included (total 5,354 participants). The institutional review board at The Catholic University of Korea approved this study (No. KCMC070T076, KC13OISI0427) and written informed consent was obtained from all participants.

### Study Protocol

Investigators were trained in the study protocol prior to carrying out physical examinations and administering the questionnaire. Detailed data on the medical history and lifestyle behavior of the subjects were obtained on the day of the investigation by interview. Data on alcohol drinking (yes/no), average alcohol amount (g/day), cigarette smoking (none, ex-smoker or current smoker), doing exercise at least once a week (yes/no) and education level were collected. Anthropometric measurements were performed with the subjects wearing light clothing, and height, weight, and waist and hip circumferences were measured according to standardized methods. Prior to the measurement of blood pressure (BP), the subjects were asked to rest for 5 min while seated. The BP on the right upper arm was measured twice and the average was recorded. Hypertension was defined on the basis of the Joint National Committee 7 report as ≥140 (systolic BP)/90 (diastolic BP) mmHg or when the subjects reported using antihypertensive medications.

### Analytical Methods and Calculations

Blood samples were collected after the subjects had fasted for at least 12 h and analyzed at a central laboratory (Samkwang Medical Laboratories, Seoul, Korea). FPG was measured using the hexokinase method and serum insulin was measured using a radioimmunoassay kit (Dainabot, Tokyo, Japan). Serum creatinine was measured using an enzymatic method. Serum total cholesterol (TC) and TG were measured using enzymatic colorimetric tests; high-density lipoprotein (HDL)-cholesterol was measured using a selective inhibition method and low-density lipoprotein (LDL)-cholesterol was calculated using the Friedewald formula. The intra- and inter-assay coefficient of variances of analytical procedures were less than 4.7% and 4.5%, respectively. In subjects with TG levels greater than 5.6 mmol/L (n = 55), the values of LDL-cholesterol were treated as missing. The homeostasis model assessment estimate of beta-cell function (HOMA-ß) and HOMA-IR were calculated using the following formulae: 20×fasting insulin (mIU/L)/[FPG (mmol/L)–3.5] and FPG (mmol/L)×fasting insulin (mIU/L)/22.5, respectively. [19]. The TyG index was calculated as the ln[fasting triglycerides (mg/dL)×FPG (mg/dL)/2] [15], [16].

### Definition of Diabetes Mellitus

In the first phase of the study, a 75 g oral glucose tolerance test (OGTT) was performed if the subject’s FPG level exceeded 6.1 mmol/L. Diabetes and prediabetes (impaired fasting glucose and/or impaired glucose tolerance) were defined according to the American Diabetes Association criteria published in 1997, which define the normal FPG level as <6.1 mmol/L [20]. In the second phase of the study, an OGTT was performed if the FPG level exceeded 5.6 mmol/L. However, from September 2009, every participant underwent an OGTT, independent of his or her FPG level. The revised report on the diagnosis of diabetes mellitus published in 2003, which set a normal FPG level of <5.6 mmol/L, was used to define the development of diabetes or prediabetes in follow-up studies [21].

### Statistical Analysis

All statistical analyses were performed using SAS 9.2 software (SAS Institute Inc., Cary, NC, USA). Data are expressed as the mean ± standard deviation (SD), as medians (25th–75th percentiles) or as proportions. For comparing the characteristics of subgroups according to the development of diabetes or TyG index quartiles, the Wilcoxon rank sum test, Chi squared test or Fisher’s exact test were used. Spearman correlation analysis was used to examine the relationships between the TyG index and other parameters. Log-Binomial regression model using Proc GenMod procedure was performed to calculate relative risk (RR), for testing the usefulness of various markers in predicting the development of diabetes. RR and 95% CI values of the upper three quartiles were calculated using the lowest quartile as the reference category. The area under the curve (AUC) of the receiver operating characteristics (ROC) curve and a 95% CI were calculated to compare the predictive power of each index. A *P* value <0.05 was considered significant.

## Results

### Baseline Characteristics

The age, body mass index (BMI), and FPG levels of the subjects were 61.6±9.3 years, 24.2±3.2 kg/m^2^, and 4.99±0.47 mmol/L, respectively (means ± SDs). Their TyG index value was 8.7±0.6, which was not significantly different between men and women (8.7±0.6 vs. 8.6±0.5; *P* = 0.191). The median (minimum, maximum) duration of follow-up was 4.6 (4.0, 8.8) years. During this period, 420 subjects (7.8%) had developed diabetes. When the baseline characteristics of these subjects were compared with those remaining free of diabetes, the subjects were found to be older, more obese and more hypertensive. FPG levels, fasting insulin levels, HOMA-IR, TC, TG and LDL-cholesterol levels were higher while HOMA-ß and HDL-cholesterol levels were lower in subjects with incident diabetes. They also had higher frequencies of a familiy history of diabetes and of alcohol drinking. The baseline values of the TyG index were significantly higher in subjects who had developed diabetes compared with nondiabetic subjects (8.9±0.6 vs. 8.6±0.6; *P*<0.0001) (Table 1).

**Table 1:** Baseline characteristics of subjects according to diabetes status at follow-up.

	Total (n = 5354)	Non-DM (n = 4934)	DM (n = 420)	*P*
Age (years)	61.6±9.3	61.5±9.4	62.9±8.6	0.007
Gender (men, %)	37.7	37.6	39.8	0.375
Height (cm)	155.8±8.6	155.8±8.6	155.7±8.4	0.927
Weight (kg)	58.8±9.7	58.7±9.7	61.0±9.9	<0.0001
BMI (kg/m^2^)	24.2±3.2	24.1±3.2	25.1±3.4	<0.0001
Waist circumference (cm)	82.7±8.6	82.4±8.5	86.3±8.6	<0.0001
Hip circumference (cm)	93.8±6.4	93.6±6.3	95.3±7.1	<0.0001
Waist–Hip Ratio	0.881 (0.839–0.924)	0.879 (0.837–0.922)	0.901 (0.861–0.947)	<0.0001
Systolic BP (mmHg)	136.4±19.0	135.9±18.7	143.1±21.1	<0.0001
Diastolic BP (mmHg)	84.5±10.7	84.2±10.6	87.5±11.4	<0.0001
FPG (mmol/L)	4.99±0.47	4.96±0.46	5.31±0.50	<0.0001
Fasting insulin (pmol/L)	35.6±31.2	35.1±30.8	41.5±35.4	<0.0001
HOMA–IR	1.1 (0.7–1.6)	1.0 (0.6–1.6)	1.4 (0.8–2.1)	<0.0001
HOMA–ß	67.3 (41.8–103.8)	67.6 (42.0–104.4)	63.4 (38.7–93.9)	0.035
Total cholesterol (mmol/L)	5.17±0.94	5.15±0.93	5.38±1.01	<0.0001
Triglycerides (mmol/L)	1.40 (0.97–2.03)	1.37 (0.96–1.99)	1.74 (1.15–2.59)	<0.0001
HDL–cholesterol (mmol/L)	1.34±0.32	1.35±0.32	1.30±0.31	0.001
LDL–cholesterol (mmol/L)	3.09±0.85	3.08±0.84	3.20±0.88	0.002
Serum creatinine (umol/L)	79.5±14.7	79.5±14.7	79.3±14.6	0.909
Hypertension (%)	24.5	23.7	34.4	<0.0001
Family history of DM (%)	8.9	8.5	13.4	0.0007
Exercise (%)	13.3	13.2	13.6	0.854
Alcohol drinking (%)	44.6	44.1	50.4	0.013
Average alcohol/day (g)	21.1±30.4	20.9±29.9	24.3±36.4	0.691
Smoking (%)				
None	70.6	70.8	67.9	0.305
Ex-smoker	12.6	12.6	12.7	
Current	16.8	16.6	19.4	
Education (%)				
None	29.4	28.8	36.9	0.008
Elementary school	49.8	50.2	44.6	
Middle school	12.3	12.4	11.3	
High school	7.0	7.1	5.3	
College	1.5	1.5	1.9	
TyG index	8.7±0.6	8.6±0.6	8.9±0.6	<0.0001

Data are expressed as means ± SD, % or median (25th–75th percentiles).

BMI, body mass index; BP, blood pressure; DM, diabetes mellitus; FPG, fasting plasma glucose; HDL, high-density lipoprotein; HOMA-ß, homeostasis model assessment estimate of beta-cell function; HOMA-IR, homeostasis model assessment of insulin resistance; LDL, low-density lipoprotein.

Next, the baseline characteristics of subjects were compared according to the TyG index quartile (Q1–Q4) groups. Subjects in the higher quartile groups were more obese, more hypertensive and had higher frequencies of smoking and alcohol drinking. The values for FPG and fasting insulin, HOMA-IR, HOMA-ß, TC, TG, LDL-cholesterol, and serum creatinine increased and the level of HDL-cholesterol decreased in proportion to the TyG index quartiles (Table 2).

**Table 2:** Baseline characteristics of subjects according to the TyG index quartiles.

	Q1 (n = 1336)	Q2 (n = 1340)	Q3 (n = 1342)	Q4 (n = 1336)	*P*
Age (years)	61.1±9.8	61.7±9.3	62.0±9.2	61.6±8.8	0.125
Gender (men, %)	37.7	38.0	35.5	39.8	0.142
Height (cm)	155.6±8.6	155.9±8.6	155.3±8.7	156.4±8.6	0.015
Weight (kg)	56.4±9.2	58.3±9.2	59.3±9.8	61.4±10.0	<0.0001
BMI (kg/m^2^)	23.3±3.1	24.0±3.2	24.5±3.1	25.0±3.1	<0.0001
Waist circumference (cm)	79.7±8.4	82.1±8.2	83.6±8.4	85.4±8.3	<0.0001
Hip circumference (cm)	92.2±6.2	93.7±6.3	94.2±6.3	94.9±6.5	<0.0001
Waist–Hip Ratio	0.860 (0.818–0.904)	0.876 (0.835–0.916)	0.887 (0.844–0.929)	0.900 (0.861–0.939)	<0.0001
Systolic BP (mmHg)	132.8±18.1	135.5±18.2	137.7±19.2	139.7±19.8	<0.0001
Diastolic BP (mmHg)	82.4±10.4	84.2±10.5	85.1±10.9	86.3±10.6	<0.0001
FPG (mmol/L)	4.82±0.48	4.96±0.45	5.02±0.43	5.14±0.47	<0.0001
Fasting insulin (pmol/L)	29.0±29.7	32.7±26.0	37.1±32.7	43.4±34.1	<0.0001
HOMA–IR	0.814 (0.511–1.243)	0.999 (0.626–1.489)	1.139 (0.713–1.671)	1.378 (0.869–2.088)	<0.0001
HOMA– ß	59.0 (36.0–92.4)	64.6 (39.8–97.7)	70.9 (45.0–106.6)	76.0 (49.3–116.8)	<0.0001
Total cholesterol (mmol/L)	4.83±0.84	5.10±0.89	5.27±0.89	5.48±1.01	<0.0001
Triglycerides (mmol/L)	0.77 (0.65–0.90)	1.17 (1.06–1.29)	1.66 (1.51–1.84)	2.68 (2.31–3.35)	<0.0001
HDL–cholesterol (mmol/L)	1.50±0.35	1.38±0.30	1.31±0.28	1.18±0.26	<0.0001
LDL– cholesterol (mmol/L)	2.97±0.76	3.17±0.82	3.19±0.83	3.02±0.95	<0.0001
Serum creatinine (umol/L)	78.5±14.2	79.4±14.5	79.3±14.5	80.8±15.6	0.001
Hypertension (%)	17.0	23.8	26.9	30.5	<0.0001
Family history of DM (%)	7.6	8.4	9.2	10.2	0.113
Exercise (%)	12.5	13.6	13.4	13.7	0.808
Alcohol drinking (%)	39.3	44.3	45.4	49.3	<0.0001
Average alcohol/day (g)	17.7±29.4	19.5±27.9	19.1±28.4	26.6±34.0	0.0004
Smoking (%)					0.007
None	72.0	71.8	72.4	66.2	
Ex-smoker	12.3	11.4	12.2	14.6	
Current	15.7	16.8	15.4	19.2	
Education (%)					0.148
None	26.7	30.1	30.4	30.4	
Elementary school	49.6	49.7	50.0	49.6	
Middle school	14.1	12.3	11.2	11.7	
High school	8.4	6.0	6.7	6.8	
College	1.2	1.8	1.7	1.4	
TyG index	8.0±0.2	8.4±0.1	8.8±0.1	9.4±0.3	<0.0001

Data are expressed as means ± SD, % or median (25th–75th percentiles).

BMI, body mass index; BP, blood pressure; DM, diabetes mellitus; FPG, fasting plasma glucose; HDL, high-density lipoprotein; HOMA-ß, homeostasis model assessment estimate of beta-cell function; HOMA-IR, homeostasis model assessment of insulin resistance; LDL, low-density lipoprotein.

### The Association between TyG Index and other Parameters

There were significant correlations between the TyG index and anthropometric indices, BP and lipid profiles. The TyG index was correlated with body weight (r = 0.185; *P*<0.0001), BMI (r = 0.215; *P*<0.0001), waist circumference (r = 0.251; *P*<0.0001), waist-hip ratio (r = 0.229; *P*<0.0001), systolic BP (r = 0.140; *P*<0.0001), diastolic BP (r = 0.145; *P*<0.0001), and with the levels of TC (r = 0.262; *P*<0.0001), TG (r = 0.984; *P*<0.0001), LDL-cholesterol (r = 0.029; *P* = 0.039), and HDL-cholesterol (r = –0.370; *P*<0.0001). As expected, the TyG index also showed significant correlations with FPG levels (r = 0.243; *P*<0.0001), fasting insulin levels (r = 0.249; *P*<0.0001), HOMA-IR (r = 0.273; *P*<0.0001), and HOMA-ß (r = 0.143; *P*<0.0001) indicating that this index could reflect the degree of insulin resistance and compensatory ß-cell function in these study subjects.

### The Incidence of Diabetes According to TyG Index Quartiles

**Figure 1:**
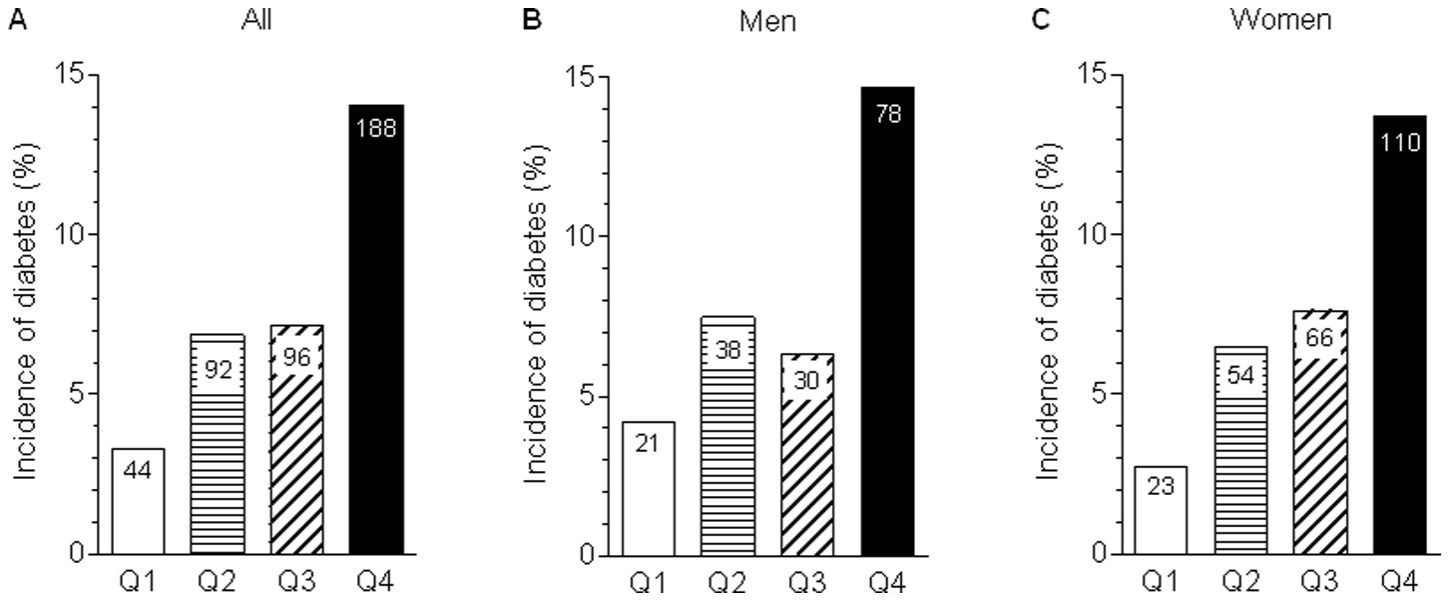
Incidence of diabetes according to the baseline TyG index quartiles in all study subjects (A), men (B), and women (C). The number in the bar indicates the number of participants with incident diabetes.

Figure 1 shows the incidence of diabetes, which increased in proportion to TyG index quartiles (Q1, 3.3%; Q2, 6.9%; Q3, 7.2%; Q4, 14.1%; *P* for trend <0.0001). Similar results could be observed in both men (Q1, 4.2%; Q2, 7.5%; Q3, 6.3%; Q4, 14.7%; *P* for trend <0.0001) and women (Q1, 2.8%; Q2, 6.5%; Q3, 7.6%; Q4, 13.7%; *P* for trend <0.0001).

### Prediction of the Development of Diabetes using the TyG Index

Log-Binomial regression analyses were performed to evaluate the TyG index in predicting the incident diabetes (Table 3). In the initial crude analysis, a statistically significant association was shown between an increased TyG index and an increased risk of incident diabetes in subjects in Q2–Q4 vs. those in Q1. Adjustment for age, gender and BMI (model 1), and further adjustment for waist circumference, systolic BP, HDL-cholesterol, family history of diabetes, smoking, alcohol drinking and education level (model 2) did not attenuate this association. Even after further adjustment for fasting serum insulin level, which reflects the degree of insulin resistance (model 3), the risk of incident diabetes was more than fourfold higher in the Q4 vs. the Q1 group (RR, 4.095; 95% CI, 2.701–6.207). There were no significant differences in the results of this analysis between men and women or between obese (BMI≥25 kg/m^2^) and nonobese (BMI<25 kg/m^2^) subjects (Tables S1, S2). To compare the predictive role of TyG index with other indices of insulin resistance, we performed similar analyses with the TG/HDL-cholesterol ratio and HOMA-IR. The TG/HDL-cholesterol ratio also proved to be a useful tool for predicting the development of diabetes, but with a significantly lower RR than the TyG index. After adjusting for age, gender, BMI, waist circumference, systolic BP, family history of diabetes, smoking, alcohol drinking, education level, and insulin, the RR (95% CI) of diabetes risk in the highest quartile vs. the lowest quartile of the TG/HDL-cholesterol ratio was 2.209 (1.566–3.115) (Table 3). Regarding the HOMA-IR values, only subjects in the highest quartile but not in the second or third quartile showed a significantly increased risk of diabetes (RR, 1.621; 95% CI, 1.171–2.243) compared with the lowest quartile group after adjustment for age, gender and BMI. However, this association disappeared after further adjustment for multiple variables (Table 3). The AUC (95% CI) of the ROC curve for the TyG index at 0.645 (0.632–0.659) was significantly higher than that of the TG/HDL-cholesterol ratio at 0.609 (0.595–0.623) or HOMA-IR at 0.592 (0.578–0.606). These results suggest that the TyG index is superior to the TG/HDL-cholesterol ratio or HOMA-IR in predicting the risk of incident diabetes.

**Table 3:** Associations of baseline TyG index, TG/HDL-cholesterol ratio and HOMA-IR with the risk of future diabetes.

	Crude	Model 1	Model 2	Model 3
TyG index				
Q1	1 (Ref.)	1 (Ref.)	1 (Ref.)	1 (Ref.)
Q2	2.163 (1.498–3.124)	2.026 (1.400–2.930)	2.099 (1.426–3.090)	2.286 (1.502–3.479)
Q3	2.261 (1.569–3.258)	2.022 (1.399–2.921)	1.960 (1.323–2.903)	2.020 (1.317–3.100)
Q4	4.806 (3.429–6.737)	4.152 (2.947–5.848)	4.098 (2.805–5.987)	4.095 (2.701–6.207)
*P* for trend	<0.0001	<0.0001	<0.0001	<0.0001
TG/HDL-cholesterol ratio				
Q1	1 (Ref.)	1 (Ref.)	1 (Ref.)	1 (Ref.)
Q2	1.528 (1.100–2.122)	1.409 (1.012–1.960)	1.422 (1.011–1.999)	1.441 (1.001–2.075)
Q3	1.598 (1.154–2.214)	1.418 (1.020–1.971)	1.308 (0.929–1.843)	1.299 (0.898–1.880)
Q4	2.847 (2.107–3.846)	2.446 (1.801–3.322)	2.267 (1.648–3.119)	2.209 (1.566–3.115)
*P* value	<0.0001	<0.0001	<0.0001	0.0002
HOMA-IR				
Q1	1 (Ref.)	1 (Ref.)	1 (Ref.)	
Q2	0.896 (0.635–1.266)	0.864 (0.610–1.223)	0.845 (0.589–1.212)	
Q3	1.269 (0.921–1.749)	1.152 (0.827–1.604)	1.084 (0.757–1.552)	
Q4	1.986 (1.474–2.676)	1.621 (1.171–2.243)	1.542 (0.997–2.387)	
*P* value	0.0001	0.015	0.103	

Results are expressed as RRs (95% CI).

Model 1: Adjusted for age, gender, and BMI.

Model 2: Adjusted for model 1+ waist circumference, systolic BP, HDL-cholesterol, family history of diabetes, smoking, alcohol drinking, and education level.

For the TG/HDL-cholesterol ratio, the HDL-cholesterol level was removed as a covariable.

For the HOMA-IR, the TG level was added as a covariable.

Model 3: Adjusted for model 2+ insulin level.

HDL, high-density lipoprotein; HOMA-IR, homeostasis model assessment of insulin resistance; TG, triglycerides.

## Discussion

In this large prospective cohort study, we found that individuals in the highest baseline quartile of TyG index had a fourfold higher risk of developing diabetes than those in the lowest quartile during a median of 4.6 years of follow-up. The predictive value of the TyG index was better than other indices of insulin resistance such as HOMA-IR or TG/HDL-cholesterol ratio. These findings highlight the usefulness of this simple index for identifying individuals with a high risk of developing diabetes.

Prospective studies on the natural history of type 2 diabetes indicate that insulin resistance is a primary defect that can occur many years before the diagnosis [17], [18]. Thus, examining the degree of insulin resistance with an accurate measure will be a key factor to improve prediction of incident diabetes. Because the hyperinsulinemic–euglycemic insulin clamp technique, a gold standard method for quantifying the degree of insulin resistance, is difficult to apply in clinical practice, several indices derived from the OGTT have been evaluated. Calculated indices reflecting insulin resistance or both insulin resistance and insulin secretory capacity showed their substantial ability to predict future type 2 diabetes [22]–[24]. However, simpler indices with a single blood test would be appropriate for a screening tool in large epidemiologic studies.

The TyG index, the product of FPG and TG levels, was proposed by Guerrero-Romero et al. as a surrogate of insulin resistance with a Pearson’s correlation coefficient of 0.322 with HOMA-IR, and –0.681 with M rates measured by the hyperinsulinemic–euglycemic clamp test [15], [16]. This correlation was similar between men and women, nonobese and obese, and nondiabetic and diabetic individuals. Another study in a Brazilian population supported this correlation [25]. The TyG index was also significantly correlated with insulin-stimulated glucose uptake measured as the steady-state plasma glucose concentration during insulin suppression testing [26], and was better associated with carotid atherosclerosis than HOMA-IR [27]. However, there are no data examining whether this index has a predictive role in identifying individuals with a high risk of developing diabetes. Our data suggest that it has a good performance in this regard. Even after adjusting for several known risk factors of diabetes and insulin level, the association between the baseline TyG index and the risk of incident diabetes was statistically significant, indicating that the TyG index is an independent risk factor. Of note, this association was similar between obese and nonobese subjects, and between men and women suggesting that the TyG index might be applicable to a wide range of subjects. The incidence rate was approximately twofold higher in subjects in the Q2 and Q3 TyG groups and more than fourfold higher in subjects in the Q4 group in this study population.

The TG/HDL-cholesterol ratio is another surrogate measure of insulin resistance demonstrated by several researchers although the relationship might differ by ethnicity [28], [29]. This simple index also has been shown to be associated with the risk of future diabetes [30], [31]. Our analysis shows that the subjects in the highest quartile of the TG/HDL-cholesterol ratio had a lower RR of diabetes development when compared with those in the Q4 TyG index group. Similarly, the RR of incident diabetes among subjects in the highest quartile of the HOMA-IR was significantly lower than for those in the Q4 TyG index group. This observation might be explained by the fact that muscle is the major organ of insulin action and glucose uptake, accounting for 85–90% of the impairment in total body glucose disposal in patients with type 2 diabetes [18], [32]. Because elevation of TG levels in blood and skeletal muscle interfere with glucose metabolism in muscle [33], the TyG index seems to mainly reflect muscle insulin resistance while HOMA-IR mainly mirrors hepatic insulin resistance [34]. Therefore, it is possible that the TyG index is superior to previously known indices for diabetes prediction, although our data need to be confirmed in other ethnic populations with different characteristics.

The strength of this study is that the study subjects were from a large scale cohort with long-term follow-up and there were sufficient cases for rigorous analysis. Diagnosis of diabetes by OGTT, although not performed in all participants in the present study, minimizes the number of overlooked cases with isolated post-load hyperglycemia. To our knowledge, this is the first report on the TyG index in an Asian population that has examined its ability as a predictive marker of future diabetes. This study also has limitations. Because the rate of follow-up loss was relatively high, the potential for selection bias needs to be considered. In addition, the findings of our study might not be generalized because it was performed in a semirural area on subjects with similar lifestyles. Although lack of information on the use of lipid-lowering agents might have influenced the result, we think that it would be minimal because only 2.3% of subjects reported that they were diagnosed as having hyperlipidemia. Another limitation is that different criteria for the normal FPG level were used at the baseline and follow-up studies.

It will be interesting to test whether a postprandial TyG index might also have meaningful effects. Because elevated postprandial levels of TG and glucose represent metabolically abnormal responses that reflect insulin resistance [35], [36], higher risks of diabetes or cardiovascular events might be associated with a higher postprandial TyG index, which remains to be clarified.

In conclusion, we propose that the TyG index is a valuable marker reflecting the degree of insulin resistance and predicting the risk of future diabetes in both men and women, and in obese and nonobese individuals. Because it can be easily calculated from routine laboratory data, our findings suggest the possibility of applying this index in risk assessment for diabetes in real clinical practice or large epidemiologic studies. Therefore, confirmation of our data in other populations is warranted for further application of this marker.

## Supporting Information

Table S1 **Association of baseline TyG index with the risk of future diabetes in men and women.**
(DOCX)

Table S2 **Association of baseline TyG index with the risk of future diabetes in nonobese and obese subjects.**
(DOCX)
