# Supplementary material for: Predicting the Development of Diabetes Using the Product of Triglycerides and Glucose: The Chungju Metabolic Disease Cohort (CMC) Study
Source: PLoS One. 2014 Feb 28;9(2):e90430. doi: 10.1371/journal.pone.0090430 (PMC3938726; doi:10.1371/journal.pone.0090430)
Supplement: Table S1 — Association of baseline TyG index with the risk of future diabetes in men and women. (DOCX) [file pone.0090430.s001.docx]

**Table S1.** Association of baseline TyG index with the risk of future diabetes in men and women.

|  | Crude | Model 1 | Model 2 | Model 3 |
| --- | --- | --- | --- | --- |
| Men |  |  |  |  |
| Q1 | 1 (Ref.) | 1 (Ref.) | 1 (Ref.) | 1 (Ref.) |
| Q2 | 1.855 (1.073–3.209) | 1.852 (1.069–3.209) | 1.888 (1.061–3.362) | 2.125 (1.124–4.015) |
| Q3 | 1.547 (0.873–2.742) | 1.510 (0.845–2.696) | 1.353 (0.731–2.506) | 1.532 (0.781–3.005) |
| Q4 | 3.951 (2.400–6.505) | 3.890 (2.315–6.539) | 3.463 (1.959–6.122) | 3.807 (2.022–7.168) |
| *P* for trend | <0.0001 | <0.0001 | <0.0001 | <0.0001 |
| Women |  |  |  |  |
| Q1 | 1 (Ref.) | 1 (Ref.) | 1 (Ref.) | 1 (Ref.) |
| Q2 | 2.445 (1.486–4.022) | 2.205 (1.337–3.637) | 2.302 (1.362–3.891) | 2.403 (1.370–4.214) |
| Q3 | 2.092 (1.787–4.711) | 2.504 (1.536–4.082) | 2.554 (1.512–4.315) | 2.438 (1.383–4.298) |
| Q4 | 5.575 (3.517–8.837) | 4.612 (2.892–7.354) | 4.782 (2.845–8.038) | 4.443 (2.530–7.802) |
| *P* for trend | <0.0001 | <0.0001 | <0.0001 | <0.0001 |

Results are expressed as RRs (95% CI).

Model 1: Adjusted for age, gender, and BMI.

Model 2: Adjusted for model 1 + waist circumference, systolic BP, HDL-cholesterol, family history of diabetes, smoking, alcohol drinking, and education level.

Model 3: Adjusted for model 2 + insulin level.
