# Supplementary material for: Predicting the Development of Diabetes Using the Product of Triglycerides and Glucose: The Chungju Metabolic Disease Cohort (CMC) Study
Source: PLoS One. 2014 Feb 28;9(2):e90430. doi: 10.1371/journal.pone.0090430 (PMC3938726; doi:10.1371/journal.pone.0090430)
Supplement: Table S2 — Association of baseline TyG index with the risk of future diabetes in nonobese and obese subjects. (DOCX) [file pone.0090430.s002.docx]

**Table S2.** Association of baseline TyG index with the risk of future diabetes in nonobese and obese subjects.

|  | Crude | Model 1 | Model 2 | Model 3 |
| --- | --- | --- | --- | --- |
| Nonobese (BMI < 25 kg/m^2^) | |  |  |  |
| Q1 | 1 (Ref.) | 1 (Ref.) | 1 (Ref.) | 1 (Ref.) |
| Q2 | 2.047 (1.277–3.282) | 1.969 (1.226–3.162) | 1.923 (1.175–3.146) | 2.080 (1.210–3.576) |
| Q3 | 2.273 (1.415–3.653) | 2.163 (1.341–3.490) | 1.914 (1.149–3.186) | 2.039 (1.163–3.576) |
| Q4 | 4.904 (3.165–7.599) | 4.671 (2.995–7.283) | 4.092 (2.506–6.681) | 4.120 (2.383–7.122) |
| *P* for trend | <0.0001 | <0.0001 | <0.0001 | <0.0001 |
| Obese (BMI ≥ 25 kg/m^2^) | |  |  |  |
| Q1 | 1 (Ref.) | 1 (Ref.) | 1 (Ref.) | 1 (Ref.) |
| Q2 | 2.139 (1.182–3.871) | 2.084 (1.150–3.776) | 2.324 (1.233–4.381) | 2.514 (1.280–4.940) |
| Q3 | 1.958 (1.092–3.508) | 1.898 (1.057–3.409) | 2.005 (1.068–3.765) | 1.955 (0.993–3.848) |
| Q4 | 3.961 (2.299–6.826) | 3.825 (2.211–6.618) | 4.285 (2.330–7.883) | 4.176 (2.168–8.044) |
| *P* for trend | <0.0001 | <0.0001 | <0.0001 | 0.0003 |

Results are expressed as RRs (95% CI).

Model 1: Adjusted for age, gender, and BMI.

Model 2: Adjusted for model 1 + waist circumference, systolic BP, HDL-cholesterol, family history of diabetes, smoking, alcohol drinking, and education level.

Model 3: Adjusted for model 2 + insulin level.
